# Design considerations for *C9orf72* disease prevention trials

**DOI:** 10.1093/brain/awaf290

**Published:** 2025-08-05

**Authors:** Michael Benatar, Adam M Staffaroni, Joanne Wuu, Michael P McDermott, Melanie Quintana, Jean Swidler, Gail Andersen, Edward D Huey, Martin R Turner, Eric A Macklin, James D Berry, Corey T McMillan, Tania Gendron, Chiadi Onyike, Howard Rosen, Hilary W Heuer, Anne-Laure Grignon, Kuldip D Dave, Calaneet Balas, Amanda Gleixner, Andrew Satlin, Billy Dunn, Penny Dacks, Adam L Boxer

**Affiliations:** Department of Neurology and the ALS Center, Miller School of Medicine, University of Miami, Miami, FL 33129, USA; Department of Neurology, University of California San Francisco, San Francisco, CA 94158, USA; Department of Neurology and the ALS Center, Miller School of Medicine, University of Miami, Miami, FL 33129, USA; Department of Biostatistics and Computational Biology, University of Rochester School of Medicine and Dentistry, Rochester, NY 14642, USA; Department of Neurology, University of Rochester School of Medicine and Dentistry, Rochester, NY 14642, USA; Berry Consultants, Austin, TX 78746, USA; Genetic ALS & FTD: End the Legacy, Philadelphia, PA 19107, USA; The Association for Frontotemporal Dementia, King of Prussia, PA 19406, USA; Department of Psychiatry and Human Behavior, Alpert Medical School of Brown University, Providence, RI 02906, USA; Nuffield Department of Clinical Neurosciences, University of Oxford, Oxford OX3 9DU, UK; Departments of Neurology and Medicine, Massachusetts General Hospital, Harvard Medical School, Boston, MA 02145, USA; Sean M. Healey and AMG Center for ALS, Massachusetts General Hospital, Boston, MA 02114, USA; Department of Neurology, University of Pennsylvania Perelman School of Medicine, Philadelphia, PA 19104, USA; Department of Neuroscience, Mayo Clinic, Jacksonville, FL 32224, USA; Department of Psychiatry and Behavioral Sciences, Johns Hopkins University School of Medicine, Baltimore, MD 21205, USA; Department of Neurology, University of California San Francisco, San Francisco, CA 94158, USA; Department of Neurology, University of California San Francisco, San Francisco, CA 94158, USA; Department of Neurology and the ALS Center, Miller School of Medicine, University of Miami, Miami, FL 33129, USA; The ALS Association, Arlington, VA 22209, USA; The ALS Association, Arlington, VA 22209, USA; The Association for Frontotemporal Dementia, King of Prussia, PA 19406, USA; Transposon Therapeutics Inc., New York, NY 92122, USA; The Michael J. Fox Foundation for Parkinson’s Research, New York, NY 10120, USA; The Association for Frontotemporal Dementia, King of Prussia, PA 19406, USA; Department of Neurology, University of California San Francisco, San Francisco, CA 94158, USA

**Keywords:** amyotrophic lateral sclerosis (ALS), frontotemporal dementia (FTD), pre-symptomatic, phenoconversion, biomarker, regulatory considerations

## Abstract

The idea that it might be possible to prevent some forms of amyotrophic lateral sclerosis and frontotemporal dementia has finally come of age. The hexanucleotide repeat expansion in the *C9orf72* gene accounts for ∼10% of all amyotrophic lateral sclerosis and 10%–15% of all frontotemporal dementia diagnoses, with the two clinical syndromes co-manifesting in a significant number of patients. As a result, clinically unaffected carriers of pathogenic *C9orf72* repeat expansions are currently the largest identifiable population at significantly elevated risk for both amyotrophic lateral sclerosis and frontotemporal dementia, and in whom it might be possible to prevent the emergence of clinically manifest disease. Strategies for the design of disease prevention trials among clinically unaffected *C9orf72* carriers have begun to emerge separately in the amyotrophic lateral sclerosis and frontotemporal dementia fields. However, recognition of the need to define neurodegenerative diseases based on biology underscores the need to consider all potential clinical manifestations of a *C9orf72* repeat expansion together, rather than the traditional siloed approach of focusing on only amyotrophic lateral sclerosis or only frontotemporal dementia. Indeed, emerging clinical and biological markers that might be used to quantify pre-symptomatic disease progression and to predict the short-term risk of phenoconversion to clinically manifest disease are shared across the phenotypic spectrum.

Given the anticipated progress in the development of therapeutic strategies to target the *C9orf72* repeat expansion, and the enthusiasm for prevention trials among the unaffected *C9orf72* repeat expansion carrier population, now is the time to begin work on the design of disease prevention trials. To this end, The Association for Frontotemporal Degeneration and The ALS Association supported a multi-stakeholder workshop (in Washington D.C., June 2024) to unify efforts to design a prevention trial for the population at elevated genetic risk for the phenotypic spectrum of *C9orf72* disease. Here we describe recommendations emanating from this workshop for the selection of outcome measures, delineation of eligibility criteria, optimal use of biomarkers and digital health technologies, potential analytic frameworks and relevant regulatory considerations related to *C9orf72* disease prevention trials. We also emphasize the importance of the amyotrophic lateral sclerosis and frontotemporal dementia communities working together in partnership with the *C9orf72* repeat expansion carrier community, the regulatory authorities and the broader drug development community.

## Teaser

Benatar *et al.* outline key recommendations from a multi-stakeholder workshop aimed at building consensus across the ALS and FTD communities on the design of a prevention trial for people at elevated genetic risk of *C9orf72*-associated disease.

## Introduction

The most common genetic cause of amyotrophic lateral sclerosis (ALS) and frontotemporal dementia (FTD) in populations of European ancestry is a hexanucleotide repeat expansion in the chromosome 9 open reading frame 72 (*C9orf72*) gene.^1,2^ In the United States, *C9orf72* mutations account for ∼10% of ALS and 10%–15% of FTD diagnoses, including ∼30%–40% of familial ALS^3^ and ∼50% of familial FTD,^4^ as well as many individuals with both FTD and ALS. Behavioural variant (bvFTD) is the most common frontotemporal clinical syndrome associated with a *C9orf72* repeat expansion. A variety of less common phenotypes—including primary progressive aphasia (PPA),^5^ neuropsychiatric syndromes^6^ and movement disorders^7,8^—have also been associated with *C9orf72* mutations, and it is currently impossible to predict the phenotype or the age of symptom onset for an individual *C9orf72* mutation carrier.^9,10^ Moreover, the underlying biological mechanisms of *C9orf72-*related neurodegeneration are thought to be similar despite the variability in associated phenotypes, with findings from animal and cell culture models believed to be equally relevant to all phenotypes.^11^ Notwithstanding progress in understanding the pathobiology of *C9orf72*, recent clinical trials of *C9orf72-*targeted antisense oligonucleotides (ASOs) as well as other small molecules have not yielded clinical benefits^12,13^—this despite these trials providing biological proof-of-concept that expression of protein products of the *C9orf72* hexanucleotide expansion can be suppressed in clinically manifest mutation carriers.^12,13^ By analogy to other neurodegenerative diseases including *SOD1* ALS and Alzheimer's disease (AD), it may be necessary to start treatment at earlier symptomatic^14,15^ or, ideally, at presymptomatic stages of disease to maximize therapeutic efficacy.

**Figure 1:**
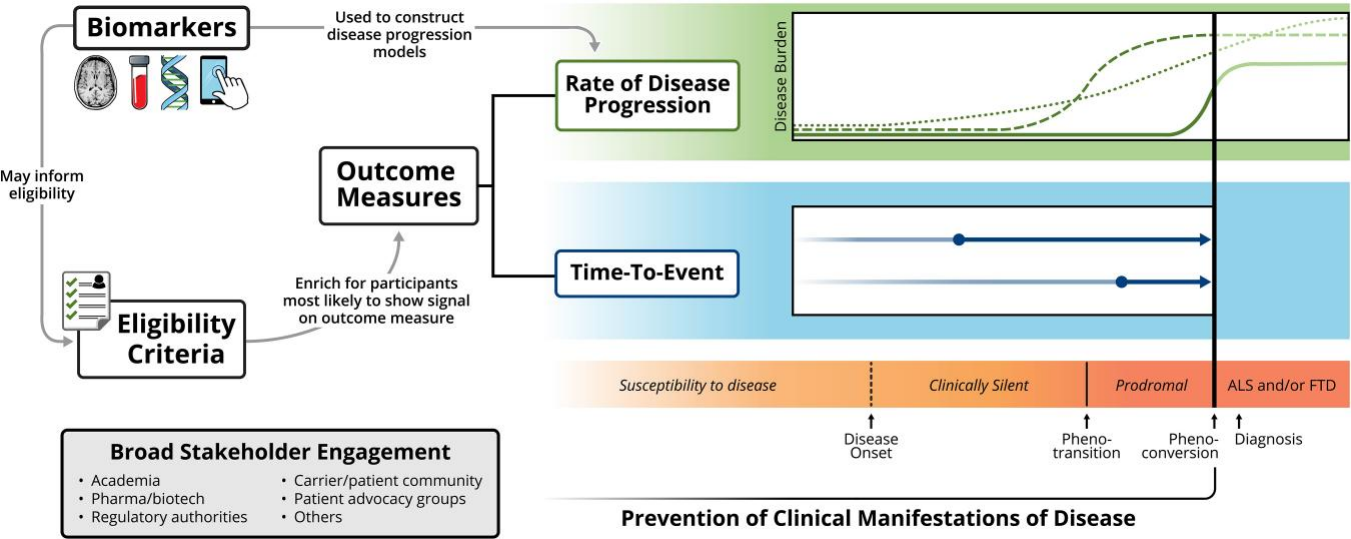
**Design considerations for *C9orf72* disease prevention trials**. The principles discussed at the C9orf72 FTD/ALS prevention trial workshop held in Washington D.C., June 2024, although centred around *C9orf72*, are more broadly applicable and summarized schematically. The orange-shaded banner (*right*, *bottom*) conceptualizes the natural history of disease as evolving through a continuum from clinically silent to clinically manifest disease (e.g. ALS and/or FTD). This paper focuses on preventing the onset of clinical manifestations of disease (rather than the onset of biologically defined disease). Outcome measures may be time to an event (e.g. phenoconversion) or rate of disease progression, which might be quantified by disease progression models that incorporate biomarkers or clinical markers of disease burden. Filled circles in the blue shaded area (*right*, *middle*) indicate the time of enrollment in a prevention trial. Dotted, dashed and solid curves in the green shaded area (*right*, *top*) depict different timings and rates of change in potential measures (or composite measures) of disease burden. Biomarkers (e.g. thalamic volume on brain MRI, neurofilament light chain concentration in blood, characteristics of the *C9orf72* repeat expansion, digital health technology-based measures) may also be used to inform eligibility criteria and thereby enrich for a trial population in whom a signal on the selected outcome measure(s) is most likely to be measurable. Importantly, broad engagement of stakeholders, including the pre-symptomatic carrier community, is essential to advancing the goals of *C9orf72* disease prevention. ALS = amyotrophic lateral sclerosis; FTD = frontotemporal dementia.

In contemplating the possibility of preventing ALS and FTD,^16,17^ it is important to differentiate strategies that aim to prevent the ‘onset of biologically-defined disease’ from those that aim to prevent ‘clinical manifestations of disease’, irrespective of whether there is evidence that the biological processes underpinning disease are already active (Fig. 1). In this manuscript we use the term ‘disease prevention’ to refer to the latter. (Parenthetically, this might be identified as either primary or secondary prevention according to the nosology of Lee *et al.*,^18^ with the distinction between the two only possible once biomarkers of disease pathology become available.) In the same vein, in the ongoing ATLAS trial, unaffected carriers of highly penetrant *SOD1* variants associated with rapidly progressive motor neuron disease and whose blood neurofilament light chain (NfL) level rises above a pre-defined threshold, are randomized to receive tofersen (a *SOD1* ASO) or placebo.^19^ Such use of NfL as a criterion for randomization depends on its established utility as a susceptibility/risk biomarker^20^—namely, that it predicts the likely timing of phenoconversion at an individual level.^21^

With anticipated progress in the development of therapies targeting genetic causes of ALS and FTD and the enthusiasm for prevention trials in the unaffected *C9orf72* repeat expansion carrier population, now is the time to begin work on the design of a prevention trial in this population. To this end, and with the support of both The Association for Frontotemporal Degeneration (AFTD) and the ALS Association (ALSA), we convened a multi-stakeholder workshop (in Washington D.C., June 2024) to lay the foundation for a *C9orf72* prevention trial. Workshop participants included community members from *C9orf72* families, ALS and FTD physicians and researchers, as well as those with clinical trial, regulatory, and biopharma expertise. This manuscript emerged from, and built upon, discussions at the workshop.

## Community perspective

Inherited diseases impacting generations can instil fear and desperation for those individuals who may be at risk, having observed the suffering and deaths of their parents and other family members. This is often the situation for people at risk for *C9orf72* repeat expansion-related clinical syndromes, underscoring the carrier community's desire that the scientific community shift towards a biological definition of *C9orf72* disease (rather than C9-ALS or C9-FTD) and that the ALS and FTD communities work together to prevent all phenotypic manifestations of *C9orf72* disease. The *C9orf72* carrier community is excited by the prospect of interventional trials that aim to slow or stop the neurodegenerative process when a treatment is most likely to yield the greatest benefits—that is, prior to the onset of symptoms. The logistical considerations of a prevention trial should include minimizing participation burden (e.g. time away from work), minimizing (when possible) burdensome or stigmatizing surveillance, and maximizing the reward-to-risk ratio. Input about behavioural changes from a study partner may be essential for early detection of clinical manifestations of disease, but the potential impact of this type of surveillance on relationships has not been studied; needless to say, this should not be done without the informed consent of the *C9orf72* repeat expansion carriers. The involvement of the *C9orf72* carrier community at all stages of prevention trial planning and execution will be critical for success, as outlined in the US Food and Drug Administration (FDA) guidance series of Patient Focused Drug Development.^22^ Input should also be drawn from people having family experience with both ALS and FTD. Although many individuals have experienced both within their family, perceptions about treatment benefit and risk may vary when individuals have primarily experienced only one clinical phenotype.

## Drug development path

Given the nascency of neurodegenerative disease prevention trials, one might expect that an experimental therapeutic would be tested in a population with clinically manifest disease before being used pre-symptomatically. This is the path that has been followed to date in AD, ALS and Parkinson's disease. However, to the extent that earlier treatment is more likely to be effective,^23^ this traditional approach risks discarding experimental therapeutics that might be effective in a prevention paradigm but have little to no effect once the disease is established. For relatively invasive treatments or those with greater potential for serious adverse effects, preliminary evidence of safety in an affected population may be needed before administration to a pre-symptomatic (healthy) population. Repurposed treatments with clear safety profiles, on the other hand, might be more readily used in a pre-symptomatic population. In addition, a gene therapy that might render someone ineligible for a future clinical trial of a different therapeutic is better undertaken in the affected rather than the pre-symptomatic population. Ultimately, considerations around the nature of the experimental therapeutic, its safety profile and potential for drug interactions, will drive the decision whether testing should begin in a pre-symptomatic or clinically manifest population. Irrespective of the study population in which an experimental therapeutic is first evaluated, however, there are challenges associated with translating findings from animal and cellular models of disease to humans. This underscores the need for biomarkers that can span both preclinical and clinical studies and expedite pathways to obtaining human biological proof-of-concept data for novel therapies.

## Concepts and terminology

Recognition that clinically manifest ALS, FTD and other *C9orf72* phenotypes are each characterized by a pre-symptomatic stage of disease aligns with the increasing shift towards defining neurodegenerative diseases based on biology rather than clinical phenotype.^24-27^ The biology underlying these disorders exists along a continuum, with phenotype evolving from a clinically silent state (akin to preclinical AD), through a prodromal stage of mild motor, cognitive, behavioural or extrapyramidal impairment (MMI, MCI, MBI or MEPI, respectively), and finally into clinically manifest disease that meets diagnostic criteria for a defined clinical syndrome.^26^ To the extent that the emergence of these clinical phenotypes can be recognized and delineated, the terms phenotransition and phenoconversion have been proposed to mark the emergence of, respectively, a prodromal syndrome and clinically manifest disease.

## Outcome measures: time-to-event versus rate of progression

In contemplating the design of a disease prevention trial among unaffected *C9orf72* repeat expansion carriers, it is essential to focus on preventing both ALS and FTD, as well as, perhaps, other phenotypes (e.g. extrapyramidal disorders) that might be the first overt clinical manifestation of disease. A critical question is whether the primary clinical outcome of a prevention trial would be a time-to-event clinical measure (e.g. time to diagnosis, phenoconversion, or phenotransition)^17^ or a longitudinal measure of rate of progression (e.g. based on a measure of motor or cognitive function) (Fig. 1). Selection of the primary outcome may depend on the stage of development and the mechanism of action of the experimental therapeutic; the stage of the disease being targeted (e.g. clinically silent or prodromal); the availability of a biomarker that is suitably fit for purpose; the planned size of the trial; and the regulatory approval pathway being pursued (refer to the ‘Regulatory considerations’ section). Ultimately, approval will require demonstration of clinical benefit that is meaningful and relevant to patients.

### Time-to-event outcome measures for binary end points

Diagnosis might feasibly be used as an end point for a prevention trial, since diagnostic criteria have been developed for the major phenotypic manifestations of *C9orf72* disease, including ALS and bvFTD,^28^ as well as for the far less commonly associated phenotypes of PPA,^29^ corticobasal syndrome^30^ and perhaps progressive supranuclear palsy.^31^ On the other hand, there are no diagnostic criteria for progressive muscular atrophy, and diagnostic criteria for primary lateral sclerosis (PLS) require at least 2–4 years of a pure upper motor neuron syndrome,^32^ and it is not clear what diagnostic criteria should be used for a parkinsonian syndrome.^33^ Similarly, psychiatric phenotypes may not always map clearly to defined Diagnostic and Statistical Manual of Mental Disorders, Fifth Edition (DSM-5) categories. An important limitation of using diagnosis as the end point is that established criteria have traditionally been developed with sporadic forms of disease in mind, with delays of ∼12 and ∼33 months between symptom onset (the closest approximation to phenoconversion when enquiring about symptoms that began in the past) and diagnosis typical of ALS^34^ and FTD,^35^ respectively.

Phenoconversion to clinically manifest disease is an alternative end point for a pre-symptomatic intervention trial, as preventing the first emergence of a well-recognized clinical syndrome is clearly meaningful. A major challenge, however, is that phenoconversion, as the primary outcome measure, would ideally consider the emergence of any of the associated disorders (e.g. motor neuron, frontotemporal, extrapyramidal, neuropsychiatric), with each requiring its own operational definition of the earliest signs/symptoms indicative of clinical manifestation. Variable progress has been made for each of these phenotypic manifestations:

- An operational definition of ALS phenoconversion has been proposed and is being used as the primary outcome measure in ATLAS, the first ALS prevention trial,^19^ but the challenges associated with more gradually progressive forms of disease are acknowledged.
- Phenoconversion to FTD might be captured by a score of 1 on the Clinical Dementia Rating plus National Alzheimer's Coordinating Center Frontotemporal Lobar Degeneration scale (CDR^®^ plus NACC-FTLD). A challenge is that this scale may not be appropriate for use by raters who are not experts in FTD.
- The operational criteria for phenoconversion to a movement disorder phenotype are not yet well established, perhaps in part due to the rarity of an extrapyramidal syndrome as the first fully manifest phenotype among *C9orf72* repeat expansion carriers.
- For neuropsychiatric syndromes, little work has been done to characterize these phenotypic manifestations of disease, what represents a clinically manifest versus prodromal state, or to develop operational criteria for phenoconversion.

It is worth noting that phenoconversion and diagnosis may coincide (e.g. when the diagnosis of ALS is made based on the aggregate of examination findings without prior subjective report of weakness). However, there may also be a lag between phenoconversion (e.g. when the patient reports abrupt onset of weakness or a study partner first notices changes in behaviour some time prior to clinical evaluation and formal diagnosis) and diagnosis, which critically depends on the timing of a thorough clinical assessment. Even more important is the gap that currently exists between criteria for phenoconversion and diagnostic criteria for ALS.^26^ In a trial that employs phenoconversion as the primary end point, the timing of phenoconversion may be operationally defined, for example, as the time of initial subjective report of symptoms that were subsequently confirmed by clinical evaluation or, if no subjective report, the midpoint between the visit at which diagnosis was made and the preceding visits—assuming that phenoconversion events in the populations are randomly distributed throughout the visit interval. Moreover, variability in the judgment of individual investigators as to emergence of clinically manifest disease might be addressed by an independent end point adjudication committee.^19^

Apart from phenoconversion, another option for a clinical end point is the phenotransition to a prodromal state. The timing of emergence of a prodromal state is, however, even more difficult to pinpoint than phenoconversion to clinically manifest disease. For example, in the motor neuron realm, the prodromal state is usually detectable only by in-depth clinical assessment rather than self-report of symptoms; thus, the recorded timing of phenotransition almost invariably relies on the happenstance timing of a clinical assessment. Similarly, in the setting of a cognitive or behavioural syndrome, phenotransition may be characterized by noticeable changes as judged by family members or an experienced clinician, but it is challenging, if not impossible, to pinpoint when such changes began. Depending on what phenomena or constructs are considered salient, and how they are operationalized, the assignment of timing of phenotransition may hinge heavily on when an assessment is performed, or factors related to the individual's relationship status or living situation, rather than on when the phenomena of interest first emerged. Moreover, constructs like MMI, MCI and MBI are non-specific and do not always imply a transitional state preceding clinically manifest disease. To use an example from a related field, amnestic MCI with evidence of amyloid and tau is prodromal for AD but far less predictive without amyloid and tau pathology. Finally, the emergence of a prodromal state that may (or may not) represent a harbinger of a clinically manifest syndrome is less intuitively clinically meaningful than the emergence of clinically manifest disease (i.e. phenoconversion).

### Continuous (or numerical) outcome measures of disease progression

Continuous measures may offer greater power to detect treatment effects, a critical consideration in rare disease trial design targeted at the presymptomatic phase, when clinical changes are expected to be subtle. Simulations in preclinical AD, for example, indicate that continuous outcomes greatly improve power over time-to-event outcomes.^36^ Broadly, the four potential clinical domains to be considered for semi-objective continuous measurements in a *C9orf72* prevention trial include motor neuron, extrapyramidal movement disorders, cognition and behaviour/neuropsychiatric features.

From a motor neuron perspective, the ALS Functional Rating Scale-Revised (ALSFRS-R) is the principal measure used to quantify disease progression in patients with clinically manifest ALS. It is, however, insensitive to quantifying motor dysfunction during the prodromal stage of disease. Indeed, MMI has been defined based on the combination of clinical examination and electromyographic findings,^37,38^ with limited effort (to date) to transform these findings into a continuous measure. Similarly, for extrapyramidal syndromes, scales such as the Unified Parkinson's Disease Rating Scale (UPDRS), the Progressive Supranuclear Palsy Rating Scale (PSP-RS) and Unified Huntington's Disease Rating Scale (UHDRS) might be used to capture early manifestations of a movement disorder in the clinically manifest population, but their sensitivity to detect prodromal extrapyramidal features among *C9orf72* carriers has yet to be explored.

Cognition is commonly affected in clinically manifest *C9orf72* repeat expansion carriers, with primary deficits observed in executive functioning, processing speed, speech and language, and social cognition.^39-41^ Executive functioning and processing speed may be the most commonly affected cognitive domains, with changes in performance occurring early, and evidence from other mutations and sporadic FTD suggests these domains may be affected regardless of clinical syndrome.^39-41^ Indeed, in *C9orf72* disease progression models, Trail Making Test Parts A and B are among the measures that change earliest in the disease course.^41^

Instruments for capturing early behavioural and neuropsychiatric symptoms are more limited. One scale with a long history of use in dementia clinical trials and substantial natural history data collected in *C9orf72* disease is the Neuropsychiatric Inventory (NPI). This study-partner-facing measure captures both the severity (distress level) and frequency of a range of neuropsychiatric symptoms that occur in FTLD/ALS, including delusions, hallucinations, apathy and disinhibition. However, like most dementia scales, it was developed for use in AD and does not assess many important symptoms in FTLD. An instrument commonly used in the ALS field is the Edinburgh Cognitive and Behavioural ALS Screen (ECAS), which includes a caregiver interview to screen for relevant behavioural and neuropsychiatric changes.^42^

Neuropsychiatric symptoms such as anxiety, depression, agitation and irritability are especially challenging. Their emergence may represent, for example, an individual's reaction to learning their *C9orf72* repeat expansion status or their lifelong risk for a fatal disease, rather than a neurodegenerative process. Moreover, these symptoms are sometimes transient, variable and responsive to interventions (both pharmacological and non-pharmacological) that have no impact on the neurodegenerative disease process. To fully capture early neuropsychiatric changes in people at risk for *C9orf72* disease, more sensitive behavioural and psychiatric assessments will be necessary.

Although measures capturing change within each of these domains in isolation may be useful as secondary outcomes for supporting approval, the ideal primary continuous outcome would measure a clinically meaningful construct and would be sensitive to change regardless of the specific *C9orf72* phenotype—and, therefore, reflect a change in the underlying neurodegenerative process. A more recent adaptation of the CDR plus NACC-FTLD, referred to as the CDR plus NACC FTLD-NM, includes neuropsychiatric and motor symptoms.^43^ Another approach, also inspired by the CDR framework, is the Multidomain Impairment Rating (MIR), which adds visuospatial and neuropsychiatric domains and incorporates motor findings.^44^ Preliminary experience with both the FTLD-CDR NM and MIR suggests that both scales more accurately capture early disease across the spectrum of genetic FTLD (personal communication), although neither has been validated in the pre-symptomatic population. Importantly, these CDR-based scales heavily weight cognition and behaviour,^45^ typically collapsing all motor manifestations (motor neuron and extrapyramidal) into a single domain.

## Biomarkers

Biomarkers potentially relevant to a *C9orf72* disease prevention trial include, to date, blood NfL, CSF dipeptide repeat proteins (DPRs), CSF and blood TDP-43 cryptic peptides and brain volumetric measures based on MRI. New proteomic^46^ and extracellular vesicle-based discovery tools have identified other biomarker candidates, though these have yet to be fully validated in at-risk *C9orf72* populations.^47^ Other promising candidates, e.g. TDP-43 seed amplification assays,^48^ will almost certainly emerge.

### Neurofilament light chain

Blood-based measurement of NfL has emerged as a biomarker with multiple contexts of use in clinically manifest ALS, including prognostic, monitoring, response and safety.^13,49-51^ Among carriers of highly penetrant pathogenic variants in *SOD1*, NfL also has value as a susceptibility/risk biomarker, predicting the short-term risk of phenoconversion to clinically manifest ALS.^19,21^ In the clinically manifest FTD population, NfL is most elevated among *GRN* carriers, intermediate among *C9orf72* repeat expansion carriers and lowest among those with a *MAPT* pathogenic variant^52-54^; baseline values appear to predict future functional decline, supporting a prognostic context of use.^54,55^ Limited data suggest longitudinal stability, but the lack of effective FTD therapeutics has precluded the evaluation of NfL as a response marker, let alone as a candidate surrogate. NfL may also have value as a susceptibility/risk biomarker in FTD, but the data are less clear than in ALS. Longitudinal data from carriers with measurements both before and after phenoconversion have not yielded a clear answer,^52,54,56-58^ but disease progression models that incorporate observations from both unaffected carriers and those with clinically manifest disease suggest that NfL concentrations at a group level may rise above expected levels in the decade prior to phenoconversion, with some individuals displaying elevations even earlier.

### Dipeptide repeat proteins

Non-ATG repeat-associated translation of DPRs in *C9orf72* expansion carriers has been observed in brain tissue many decades before the emergence of symptoms and is detectable in CSF. Higher DPR levels are apparent in *C9orf72* repeat expansion carriers compared to controls but show no clear relationship to clinical outcomes.^59-61^ There have been two early phase trials of expansion-targeting therapy in symptomatic *C9orf72*-related ALS and FTD that successfully lowered levels of CSF DPRs but failed to lower NfL levels.^13,62^ CSF DPR concentration is similar between those who are pre-symptomatic and those with clinically manifest disease.^59-61^ As such, there is no reason to expect that DPRs will be useful in predicting phenoconversion to clinically manifest disease. Whether DPRs may still provide a useful measure of pharmacodynamic effect in the unaffected carrier population is unclear, given the disconnect observed in the clinically manifest population between DPR reduction and therapeutic effect.

### TDP-43 cryptic peptides

TDP-43-dependent cryptic peptides, including the cryptic form of HDGFL2 among others, have emerged as potential biomarkers for ALS and FTD, reflecting loss of TDP-43 function consequent to its aggregation and phosphorylation in the cytoplasm along with TDP-43 nuclear depletion.^63,64^ Preliminary studies in both CSF and plasma have largely relied on cross-sectional samples collected from patients with clinically manifest ALS and individuals at elevated genetic risk for ALS. The concentration of cryptic HDGFL2 peptide in CSF was elevated in about a third of *C9orf72* repeat expansion carriers, irrespective of whether they were in the pre-symptomatic or clinically manifest stage of disease. A similar proportion of patients with sporadic ALS were found to have elevated levels of cryptic HDGFL2 in CSF. Moreover, the concentration of cryptic HDGFL2 was generally higher among those with shorter disease duration at the time of sampling than among those with longer disease duration, leading to the hypothesis that cryptic HDGFL2 levels may peak pre-symptomatically, and then decrease during clinically manifest disease. A clearer understanding of the temporal course of cryptic HDGFL2 in pre-symptomatic disease and its potential use as a susceptibility/risk biomarker predicting phenoconversion to ALS or FTD will require more sensitive cryptic HDGFL2 assays and longitudinal measures of cryptic HDGFL2 in samples obtained pre- and post-conversion.

### Neuroimaging

There is mounting evidence for structural MRI differences between pre-symptomatic carriers and controls^65-68^ at the group level. Interpretation of these results, however, is complicated by the variety of methodological approaches used (e.g. cortical thickness and volumetric measures) and the inconsistent definition of pre-symptomatic disease (e.g. CDR plus NACC FTLD = 0^66^ versus a normal neuropsychological and neurological exam at MRI^67^ versus lack of clinician-rated progression^68^). Moreover, early neuroimaging evidence is frequently FTD- or ALS-centric, lacking comprehensive considerations of the presence of prodromal FTD and/or ALS early clinical features. Nonetheless, a consistent feature of these studies in both ALS and FTD is that cortical regions such as the insula and deep grey matter structures such as the thalamus appear to hold the most promise as pre-symptomatic markers, with some utility in predicting the timing of phenoconversion.^69^ The latter observation is consistent with the known early formation of DPR inclusions in the thalamus^70^ and MRI studies using *C9orf72* promotor methylation as a proxy for *C9orf72* expression that demonstrate correlations between the methylation and thalamic volume in clinically manifest FTD and ALS.^71^ Moreover, thalamic volume has been incorporated into FTD disease progression models to estimate disease age—a quantifiable measure of years to symptom onset—and the models estimate that thalamic volumes in carriers deviate from those of non-carrier controls as early as 3–4 decades prior to symptom onset.^41^ Few longitudinal MRI studies have been reported in pre-symptomatic *C9orf72*^72-76^; it is, therefore, unclear how much this modality will contribute to monitoring longitudinal change in a prevention trial.

### Digital health technologies

Digital health technologies (DHTs) are ‘systems that use computing platforms, connectivity, software, and/or sensors for health care and related uses’.^77^ In addition to creating opportunities to capture novel streams of quantitative data informative of physical and cognitive function, DHTs may be used to capture traditional outcome measures remotely and more frequently. Remote data collection may reduce participant travel burden by facilitating visits from home, thereby enhancing recruitment, retention, diversity and inclusivity. Greater frequency of assessments may improve statistical power to detect treatment effects relative to less frequently measured traditional outcome measures.^78,79^ Broadly speaking, candidate DHTs can assess function through active engagement of the participant in a task or passive data collection as the participant goes about daily routines. Two tablet-based DHT tools have been evaluated for assessing cognition in familial FTD: GENFI Ignite and a modified version of the NIH-EXAMINER (Executive Abilities: Measures and Instruments for Neurobehavioural Evaluation and Research). GENFI Ignite is an iOS app designed as a self-assessment tool to test executive functioning, processing speed and social cognition. The NIH-EXAMINER is a laptop-based, clinician-administered battery of executive functioning and processing speed tasks, with data supporting its sensitivity to deficits in clinically manifest bvFTD.^80^ A study in familial FTD cases, including *C9orf72*, suggested that this battery could detect group-level cross-sectional and longitudinal changes in presymptomatic disease.^81^ Recently, the NIH-EXAMINER has been modified for use on the tablet, with preliminary data suggesting sensitivity to the early stages of clinically manifest familial FTD.^82^

The ALLFTD Mobile Smartphone App was designed to measure the range of clinical manifestations of FTD through cognitive testing, motor testing, collection of speech samples and passive data from the phone's sensors, including step count, battery usage and GPS (with participant opportunity to opt out of these passive data collection streams). This app has been piloted in the ALLFTD network, with initial analyses demonstrating feasibility and high acceptance in the first 214 study participants.^83^ In one study, these app-based cognitive tests produced reliable results that were associated with performance on gold standard tests, were sensitive to disease progression, and at the group level, were sensitive to statistically worse performance among prodromal mutations carriers compared to controls.^84^ In studies of clinically manifest ALS, a variety of smartphone-based platforms have been used to monitor symptom progression, with a focus on speech recordings, patient reported outcomes (PROs) of physical function, fatigue, quality of life, mood and communication, and actigraphy, using passive data collected from phone sensors.^85-89^

Voice recordings can also be analysed for acoustic and linguistic content that may be sensitive to the cognitive, motor and emotional manifestations of *C9orf72* repeat expansions. In FTD, automated analyses of speech recordings can differentiate FTD syndromes (e.g. bvFTD, non-fluent variant PPA) with high accuracy^90^ and may detect early changes in presymptomatic familial FTD at a group level.^91^ In clinically manifest ALS, features of motor speech can be extracted from speech recordings made on mobile phones or tablets and analysed to produce outcome measures that change with ALS progression.^92^ Speaking and pause rates, two easily obtained features of motor speech, correlate well with communicative participation in people with clinically manifest ALS.^93^

The number of passively collected data streams is multiplying quickly, and a comprehensive discussion was beyond the scope of the workshop and this publication. The most support exists for actigraphy data that may capture aspects of movement, such as step count, speed of movement and total quantity of movement relevant to ALS and various extrapyramidal movement disorder manifestations of *C9orf72* disease. In clinically manifest ALS, daily activity and step counts decrease as the disease progresses^94,95^; and measurements of individual limb movements may enable more granular assessment of functional change.^94,96^ For the aforementioned approaches, however, it remains unclear to what extent DHTs are sensitive to pre-symptomatic disease, have the capacity to quantify disease progression during the pre-symptomatic stage of disease, or predict phenoconversion to clinically manifest ALS and/or FTD at an individual (not just a group) level.

## Eligibility criteria

Unaffected carriers of a *C9orf72* repeat expansion are at significantly elevated risk for ALS, FTD, FTD-ALS and other phenotypes. While the risk of phenoconversion to clinically manifest disease increases with age, penetrance is incomplete with significant variability within families,^97-99^ and age of symptom onset of an unaffected carrier is not reliably predicted by the parental or average familial age of symptom onset.^9^ Moreover, the annual rate of phenoconversion is low, in line with the wide distribution of age of onset. Among *C9orf72* carriers over the age of 45, for example, unpublished data from the Pre-Symptomatic Familial ALS (Pre-fALS) study suggest that the annual rate of phenoconversion is 3%–4%. Even a trial designed to reduce phenoconversion by 50% would require a sample size of at least 500 with 5 years of follow-up if it enrolled any *C9orf72* carrier over the age of 45. Designing an adequately powered clinical trial, in which follow-up duration cannot feasibly exceed 5 years, will require that eligibility criteria enrich for a population at meaningfully higher than average risk for phenoconversion in the relatively short term (Fig. 1). Factors such as age, the presence of a prodromal syndrome (e.g. MMI), elevated NfL concentration, regional brain atrophy and personalized estimates of penetrance^99^ have been identified as potential predictors. Unlike highly penetrant *SOD1* variants associated with rapidly progressive disease in whom a rise in blood NfL levels may be sufficient to predict phenoconversion,^19,21^ predicting phenoconversion among *C9orf72* repeat expansion carriers will likely require a multimodal approach that includes a combination of these prodromal clinical markers and biomarkers.^41^ A similar multimodal approach would almost certainly be needed to predict the short-term risk of phenotransition (i.e. from clinically silent to prodromal). If a continuous measure of disease is used to evaluate treatment effect, then eligibility criteria should also enrich for a population likely to have discernible progression of disease based on this measure during the period of the trial.

## Analytical strategies

Natural history data collected from the target population of unaffected people with the *C9orf72* repeat expansion are critically important for the design of future prevention trials. Such data exist separately within the context of studies originally funded to investigate either ALS^100^ or FTD,^101,102^ although each of these initiatives has also captured individuals who have phenoconverted to FTD and ALS, respectively. Harmonization of these datasets in terms of outcomes and phenoconversion criteria that can be applied to both populations is crucial to shifting the focus from specific phenotypes to the collective *C9orf72* population. Such data would be valuable in establishing the reliability, validity and sensitivity of candidate measures in this population and determining which have the properties required of a primary outcome for a prevention trial. Trial design would also be greatly enhanced by using natural history data to define the eligibility criteria needed to select an enriched population at meaningfully greater risk for measurable decline in the primary outcome of interest during a trial.

As discussed earlier (see ‘Time-to-event outcome measures for binary end points’), a natural primary outcome could be defined as the time from randomization to phenoconversion, i.e. the appearance of clinically manifest ALS or FTD according to standardized criteria ascertained by the site investigator and adjudicated by an independent committee. The advantage of focusing on phenoconversion is that it is precisely the event that is the target of prevention. As such, it is of primary clinical relevance. Disadvantages are that the *C9orf72* repeat expansion does not have complete penetrance, phenoconversion is a rare event, and often people who phenoconvert do not yet meet diagnostic criteria for a clinical syndrome (until their disease is relatively advanced). These considerations all lead to the need for larger sample sizes. This problem could be mitigated by appropriate enrichment of the trial cohort, but that in turn could pose substantial challenges for recruitment.

Estimates of an individual's latent ‘disease age’ (i.e. estimated years to symptom onset)^41^ based on jointly modelling clinical and biomarker data as predictors have the potential to predict which individuals are approaching phenoconversion (to inform trial eligibility criteria) and which outcomes are most sensitive to change during the pre-symptomatic period. A similar approach has been used successfully in Huntington's disease.^103^ A model that predicts progression as a function of multiple pertinent assessments that index different disease stages could be used as an analysis tool to assess the slowing of disease progression across a broad *C9orf72* population.^104,105^ Model development would require data from a large cohort of participants with a *C9orf72* mutation, including harmonized variables from pre-symptomatic ALS and FTD cohorts such as neuromuscular examination, electromyography, neuropsychological testing, behavioural evaluation, neuroimaging and biofluid biomarkers. There are also lessons to be learned from the DIAN-TU-001 trial of gantenerumab and solanezumab, where disease progression model assumptions (proportionality, monotonic decline and consistent variance across symptomatic and asymptomatic participants) based on natural history data were not met in the analysis of clinical trial data.^106^

Joint models^107^ that combine multiple outcome measures also provide a method for incorporating time to phenoconversion in an analysis otherwise focused on progression based on a continuous end point. This would be especially advantageous if the treatment had discernible effects on both components of this composite end point. This approach has been recommended by the FDA for ALS clinical trials, with the ALSFRS-R and time to death being the longitudinal and event markers, respectively.^108-110^

## Regulatory considerations

Regulatory insights from relevant regional authorities, such as the FDA and European Medicines Agency (EMA), should be included at early stages of clinical trial planning. The FDA's draft guidance on drug development for the treatment of early AD^111^ is also pertinent to regulatory considerations for a *C9orf72* disease prevention trial. Unsurprisingly, since the earliest stages of AD are clinically silent, this guidance document recognizes the importance of diagnostic criteria—and therefore trial eligibility criteria—that are predicated on biology (i.e. biomarker) rather than clinical syndrome (i.e. phenotype). Notwithstanding the need for a biological approach to disease, the FDA recognizes the importance of defining study populations based on conceptual categories of AD such as stage 1 (characteristic pathophysiological changes but no clinical manifestations) or stage 2 (characteristic pathophysiological changes and subtle abnormalities detected with sensitive neuro-psychological measures or subjective complaints without functional impairment).^27^ These categories are analogous to the concepts of clinically silent and prodromal disease, as described earlier for ALS and FTD.

The FDA recommends incorporating both clinical outcome assessments (COAs) and biomarkers in pre-symptomatic and early symptomatic clinical trials, noting that the approval pathway may differ based on the defined primary end point and its ability to measure a clinically meaningful effect. In the context of phenotypic manifestations of *C9orf72* disease, outcomes should adequately represent all (major) clinical syndromes. Time-to-event (e.g. phenoconversion) is generally acceptable as a primary efficacy measure in prevention trials. Prevention trials that utilize such clinically meaningful end points will likely require longer durations and larger sample size than traditional trials conducted in a clinically manifest population. By contrast, while ‘disease age’ has been proposed as a continuous measure of disease,^41^ this approach has not been accepted by regulatory agencies at this time, and these disease progression models should be updated to ensure that motor neuron and extrapyramidal relevant aspects of disease are adequately captured.

The FDA also recognizes that biomarker surrogate end points may be needed in early intervention/prevention trials since clinical manifestations of disease may be modest or minimally progressive over the course of a trial. A therapeutic effect on a surrogate end point that is ‘reasonably likely to predict clinical benefit’ may form the basis for accelerated approval. However, while the FDA has considered the pharmacodynamic response in NfL in regulatory decisions for tofersen in the clinically manifest *SOD1* ALS population,^15,112^ the agency also has underscored the unique nature of this particular circumstance and discouraged investigators from inferring that future approval decisions will follow the same path. Ultimately, the FDA will consider all available evidence and the consistency of results across an array of end points for regulatory decisions.

The FDA has publicly supported the use of DHTs in clinical trials and has published guidance outlining what is needed for clinical validation.^77^ Depending on the context of use, a DHT can support a digitally-derived end point or a digitally-derived biomarker. If the goal is to support a digitally-derived end point, for example, all the typical COA recommendations about validation, clinical meaningfulness, and context of use also apply.^22^ Although digitally derived outcomes can be sensitive to detecting small clinical changes, their utility in trials and regulatory submissions critically depends on established clinical meaningfulness and interpretability of those changes. For example, identifying actigraphy markers that change at the onset of symptoms from *C9orf72* disease may be of limited value if the clinical relevance of these observations is not yet known. Regulators in different global regions may also have different perspectives on what evidence is needed to support the DHT data that are being collected.

## Call to action

There is great enthusiasm from the *C9orf72* carrier community for disease prevention trial(s), and significant progress has already been made by both ALS and FTD investigators working in the field. With increasingly promising therapeutics in development, the time is right to fully ready the community to design prevention trials for clinically unaffected *C9orf72* carriers. At present, the informal consensus from the workshop was that more work is needed to prepare the field to design and implement prevention trials. There was also agreement that work should begin expeditiously. Critical areas that can be addressed are as follows:

- Consensus is needed around primary outcome measures for pivotal prevention trials. The ideal clinical end points should be both measurable by clinicians and meaningful to the individuals at risk of disease. Moreover, if phenoconversion to clinically manifest disease is the key meaningful event, consensus will be needed across ALS and FTD experts on the operational definition of phenoconversion in the context of *C9orf72* disease.
- Disease progression models and the ‘disease age’ composite measure developed by FTD researchers in *C9orf72* carriers are promising tools for prevention clinical trials that should be updated to ensure that they adequately capture characteristics of motor neuron disease and extrapyramidal movement disorders.
- Emerging biomarkers of TDP-43 pathology hold promise for cohort enrichment and as response biomarkers in both ALS and FTD phenotypes. These new biomarker candidates will require both analytical and clinical validation, including in observational studies tracking *C9orf72* carriers before and after phenoconversion to clinically manifest ALS and FTD.
- Pre-symptomatic *C9orf72* carriers might be included in studies primarily focused on the affected population, depending on the specific objectives of doing so. Such an approach would require a clear plan for evaluating the impact of the experimental therapeutic in this population, which, in the absence of overt clinical manifestations of disease, would rely primarily on biomarkers. This is well illustrated by the DIAN-TU-001 trial of gantenerumab and solanezumab that included both unaffected carriers of mutations in a dominantly inherited AD gene and those with early dementia.^106^ In this study, there was no measurable progression of the primary (cognitive) end point in the asymptomatic population, but a reduction in amyloid burden on PET imaging (gantenerumab) was apparent in the asymptomatic group.
- The ALS and FTD clinical research fields must overcome longstanding silos and join forces in the effort to prevent *C9orf72* disease. Eligibility criteria, outcome measures and response biomarkers in prevention trials must be validated across multiple fields of expertise. For natural history studies, biomarker and DHT development requires ALS and FTD researchers to collaborate early and often, ensuring that the candidates developed by one field are applicable to the other. Importantly, experts from other fields (e.g. movement disorders) should also be engaged, since *C9orf72* disease is not limited to ALS and FTD phenotypes.
- There is a pressing need for a composite scale that considers all relevant clinical domains (motor neuron, extrapyramidal movement disorders, cognition and behaviour/neuropsychiatric features). Such a scale should be developed collaboratively to ensure that perspectives from different clinical communities are appropriately considered.
- Studies to assess the meaningfulness of outcome measures should include the perspectives of people with lived family experience of C9-ALS, C9-FTD and combinations thereof, so that such outcome measures can be deployed with confidence in their appropriateness for all *C9orf72* carriers.

While the selection of a therapeutic agent is obviously critical to disease prevention trials, the foregoing discussion is intentionally silent on this consideration. Rather, the goal has been to ensure that the necessary foundational work has been completed by the time promising therapeutic candidates emerge. In turn, this will accelerate the speed with which disease prevention trials, whether using a traditional or master protocol (e.g. platform or basket designs),^113^ can be initiated—thus bringing us closer to the ultimate goal of preventing *C9orf72*-related neurodegenerative disease.
